# Exploring the Origin and Relatedness of Maternal Lineages Through Analysis of Mitochondrial DNA in the Holstein Horse

**DOI:** 10.3389/fgene.2021.632500

**Published:** 2021-07-15

**Authors:** Laura Engel, Doreen Becker, Thomas Nissen, Ingolf Russ, Georg Thaller, Nina Krattenmacher

**Affiliations:** ^1^Institute of Animal Breeding and Husbandry, Christian-Albrechts-University, Kiel, Germany; ^2^Institute of Genome Biology, Leibniz Institute for Farm Animal Biology (FBN), Dummerstorf, Germany; ^3^Verband der Züchter des Holsteiner Pferdes e.V., Kiel, Germany; ^4^Tierzuchtforschung e.V. München, Grub, Germany

**Keywords:** mitochondrial DNA, Holstein horse, maternal lineages, genetic diversity, phylogeny

## Abstract

Maternal lineages are important for the breeding decision in the Holstein horse breed. To investigate the genetic diversity of the maternal lineages and the relationships between founder mares, the maternal inherited mitochondrial genome (except the repetitive part of the non-coding region) of 271 mares representing 75 lineages was sequenced. The sequencing predominantly revealed complete homology in the nucleotide sequences between mares from one lineage with exceptions in 13 lineages, where differences in one to three positions are probably caused by *de novo* mutations or alternate fixation of heteroplasmy. We found 78 distinct haplotypes that have not yet been described in other breeds. Six of these occurred in two or three different lineages indicating a common ancestry. Haplotypes can be divided into eight clusters with all mares from one lineage belonging to the same cluster. Within a cluster, the average number of pairwise differences ranged from zero to 16.49 suggesting close maternal relationships between these mares. The results showed that the current breeding population originated from at least eight ancestral founder mares.

## Introduction

The Holstein horse is one of the most popular horse breeds and especially known for its show jumping ability. Every year, the World Breeding Federation for Sport Horses (WBFSH) publishes the most successful breeds in international show jumping based on competition results. In the last years, the Holstein horse has always been ranked in the top ten jumping breeds. The maternal lineages receive special attention and are considered to be important for the breeding success in the Holstein horse. The documentation of the maternal lineages has already started at the beginning of the 19th century; at that time, each mare with unknown parents was defined as a founder mare for a new lineage. The Holstein breed originated in the marsh lands of Schleswig-Holstein, northern Germany. Formerly, five different breeding districts existed, each with its own breeding organization, which later merged. In the first district, mares were assigned to lineage numbers one to 1,000. Then, maternal lineages were successively assigned in the other four districts, with 1,000 numbers for each district. Thus, at that time the region of origin of mares can be derived up to the lineage number 5,000. After the first 1,000 numbers per district were assigned, new maternal lineages received numbers from 5,001 onward regardless of the district. In total, this resulted in more than 8,900 different lineages. However, the number of maternal lineages has decreased substantially, especially after World War II. Today, 437 maternal lineages have been preserved, but there are large differences in the number of mares per maternal lineage. The breeding population currently comprises 5,729 active brood mares (Fédération Equestre Nationale [FN], 2019). The effective population size was estimated at 55.31 individuals (Roos et al., 2015). Information about the relationships between the founder mares is limited and the history of the maternal lineages has not yet been genetically determined.

Due to its maternal inheritance, its lack of recombination and the high mutation rate (Hutchison et al., 1974), the mitochondrial DNA (mtDNA) is particularly suitable to examine maternal lineages on a molecular genetic basis. The mitochondrial genome is a circular, double-stranded and haploid molecule (Boore, 1999) with a length of 16,600 bp that contains 37 genes and only one non-coding region (Xu and Árnason, 1994). Most of the previous studies on mtDNA in horses have focused on phylogenetic issues and have already shown, that the analysis of mtDNA can be a useful tool to investigate intra- and interbreed relationships. Furthermore, the history of various horse breeds has been elucidated through mtDNA analysis (Hill et al., 2002; Kavar et al., 2002; Mahrous et al., 2017).

As mitochondrial genes are involved in energy metabolism, genetic variation on mtDNA level might contribute to lineage specific differences in performance traits. In humans, mitochondrial variation has been reported to affect endurance and power capabilities (Dionne et al., 1993; Niemi and Majamaa, 2005). In horses, research has mainly focused on racing performance. For Thoroughbreds, Harrison and Turrion-Gomez (2006) examined the whole mitochondrial genome and found haplotypes associated with racing success and Lin et al. (2018) found a mutation in the mitochondrial *16S rRNA* gene resulting in low racing performance. So far, no corresponding association studies were done for other traits or in German warmblood breeds. The Holstein horse has been selected intensively for its athletic performance and show jumping ability, which requires a high amount of energy comparable to racing performance (Piccione et al., 2013). Hence, it seems to be likely that mitochondrial variation, and thus, maternal lineages, could have an influence on performance traits in Holstein horses. Therefore, we studied Holstein maternal lineages based on mtDNA, thereby establishing a basis for further investigations.

Most of the previous studies regarding mtDNA in horses considered only the non-coding region of the mitochondrial genome because of its high variability (Aquadro and Greenberg, 1983) and use for resolving phylogenetic backgrounds of the examined breeds. However, as reported by Achilli et al. (2012) and Cardinali et al. (2016), respectively, resolution of maternal lineages can be improved by increasing the length of the sequenced fragment. Therefore, the mitochondrial genome from Holstein mares representing a substantial part of the current breeding population was considered in this study. The nucleotide variation of the mtDNA within lineages was examined to assess the intra-lineage diversity and to examine the accuracy of pedigrees. Secondly, the genetic variation between all individuals was analyzed in order to figure out the relationships between the different maternal lineages.

## Materials and Methods

A cohort of 493 mares was preselected based on the availability of genotypes (provided by an in-house project) to allow consideration of interactions between the mitochondrial and nuclear genome in further evaluations. The maternal lineage was of no interest for the in-house project. Instead, mares were selected that show a low level of preselection and a low pedigree relationship. Additionally, mares with extensive phenotypes were primarily selected in order to enable envisaged studies on the influence of mtDNA on performance traits. Only mares that were registered in the studbook since 2015 were considered and preference was given to mares whose owners lived in Germany to increase the response rate of samples. The breeders of the mares were asked to collect hair samples during routine care, e.g., combing the mane or tail. After two sampling periods in February and December 2019, hair samples from 271 mares were made available by 207 different breeders. Pedigrees for all mares can be traced back up to ten generations.

Total DNA was extracted from 20 to 25 hair roots for each animal implementing a modified protocol according to Miller et al. (1988) that contains a pre-cleaning of the samples. Briefly, 700 μl Isopropanol were added to 20–25 hair roots and incubated at room temperature for 24 h before DNA was isolated. For the amplification of the mitochondrial genome, nine overlapping primer pairs, each with a product length of approx. 2,000 bp, were generated stepwise using the software PRIMER 3 (Rozen and Skaletsky, 2000). Polymerase chain reactions (PCR) were performed in a 12 μl reaction volume containing 20 ng total DNA, 0.2 μM of the forward and reverse primer, 200μM dNTPs, 1.25 U of the PrimeSTAR GXL DNA-Polymerase (Takara) and the reaction buffer supplied by the manufacturer. The amplification started with an initial denaturation at 98°C for 2 min followed by 30 cycles, each with a denaturation at 98°C for 20 s, annealing at 60°C for 15 s and an extension at 68°C for 2:30 min. After checking the success of the amplification on a 2% agarose gel, PCR products were purified with the thermosensitive alkaline phosphatase (FastAPTM, Fermentas, Sankt Leon-Rot, Germany) and Exonuclease I (Fermentas, Sankt Leon-Rot, Germany). For sequencing, two additional primers were generated for each PCR product, resulting in 36 sequencing reactions for the total mitochondrial genome. Sequencing was done using the ABI 3130*xl* Genetic Analyzer and the BigDye^®^ Terminator v3.1 Cycle Sequencing Kit (Applied Biosystems, Foster City, CA, United States).

Sequences were analyzed using the software Sequencher 5.0 (Gene Codes Corporation, Ann Arbor, MI, United States) and were compared to the GenBank reference sequence X79547.1^1^. Ambiguous sequences were excluded from analysis and sequencing was repeated. The repetitive part of the non-coding region was excluded in all samples due to unsuccessful sequencing.

Parameters of genetic diversity including the number of polymorphic sites (S), nucleotide diversity (π), number of haplotypes (Nh), and the haplotype diversity (Hd) were estimated using DnaSP 6 (Rozas et al., 2017). To visualize the genetic relationship between all individuals, a principal coordinate analysis (PCoA) based on the genetic distances according to Tamura and Nei (1993) was applied. Haplogroups were assigned and named as defined by Achilli et al. (2012). The software Arlequin 3.5 was used to perform intra- and intergroup comparisons based on the mean number of pairwise differences. Pairwise *F*_ST_ values were estimated according to Weir and Cockerham (1984). The standard quantitative scale was used to evaluate *F*_ST_ values, where a value between 0 and 0.05 indicates none or low genetic differentiation; a value between 0.05 and 0.15 moderate differentiation; a value between 0.15 and 0.25 high differentiation; and values above 0.25 very high genetic differentiation. The evolutionary relationships between haplotypes were visualized by a median joining network constructed with PopArt 1.7 software (Leigh and Bryant, 2015). The black circles illustrate the haplotypes whereas the size of the circles is proportional to the haplotype frequency, i.e., the number of individuals belonging to each haplotype. The small circles shown in red are called median vectors (mv) and represent hypothesized haplotypes that do not exist in the examined sample and are intended to connect the present haplotypes with each other. The strokes between two haplotypes indicate the number of mutations.

## Results

**FIGURE 1:**
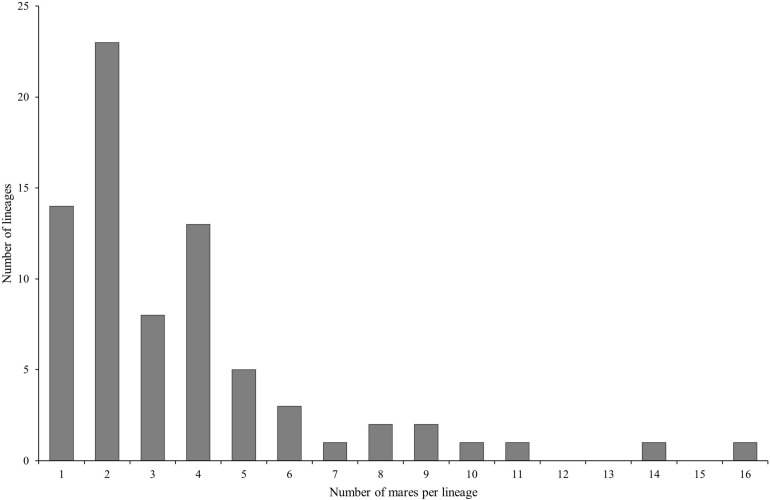
Distribution of the number of mares per lineage in the dataset (*n* = 271 Holstein mares belonging to 75 different lineages).

Hair samples from 493 mares were requested. We received samples from 271 mares which correspond to a response rate of 54.97%. At sampling time, the mean age of the mares was 6.25 ± 2.99 years. The mares belong to 75 lineages, i.e., 17.4% of all registered maternal lineages of the current breeding population. The number of samples per maternal lineage was 3.61 on average and ranged between one and 16; the majority of the lineages (77.33%) was represented by one to four mares (Figure 1).

**FIGURE 2:**
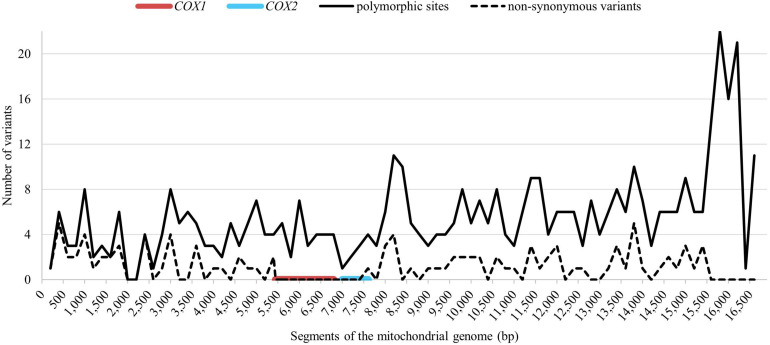
Distribution of the total polymorphic sites (solid line) and the non-synonymous substitutions (dashed line) across the mitochondrial genome. The red line indicates the conserved region around COX1 (EquCab3.0, MT:5,362–6,906) and the blue line indicates the conserved region around COX2 gene (EquCab3.0, MT:7,048–7,731).

The mitochondrial genome from all 271 representatives of 75 Holstein maternal lineages was sequenced and compared to the GenBank reference sequence (X79547.1). In total 467 polymorphic sites were identified, 11 of those were indels, the other 456 were single nucleotide polymorphisms (SNPs). So far, 354 polymorphic sites have not yet been reported in other breeds, according to mtDNA sequences which are available in the NCBI database. Of that, 22 polymorphic sites occurred in all examined Holstein mares. Figure 2 shows the distribution of the polymorphic sites across the mitochondrial genome. The nucleotide positions were numbered according to Xu and Árnason (1994). 381 polymorphic sites were located in the coding region making of 2.46% of all sites in that region. A higher variability could be observed in the non-coding region with 86 polymorphic sites corresponding to 7.60% of the non-coding sites. There were 103 non-synonymous substitutions (see dashed line in Figure 2). No non-synonymous substitutions could be observed in the section between bp 5,499 and 7,599 covering the complete *COX1* gene and 552 bp of the *COX2* gene (80.67%).

To determine the diversity within lineages, the mtDNA sequences of all mares within a lineage were compared regarding their polymorphic sites. For this purpose, data from 14 lineages with only one mare were excluded from the analysis. There were no within-lineage differences between mtDNA sequences in 50 of the remaining 61 lineages. In 11 lineages, the mares’ mtDNA sequences differed in one to three sites. A more detailed analysis of the pedigrees of the mares did not shed light on the observed nucleotide differences: in nine lineages, mares had a common female ancestor one to seven generations ago, but in four lineages no common female ancestor could be found in the previous ten generations. There was no association between the number of nucleotide differences and the number of generations up to the first common female ancestor.

**FIGURE 3:**
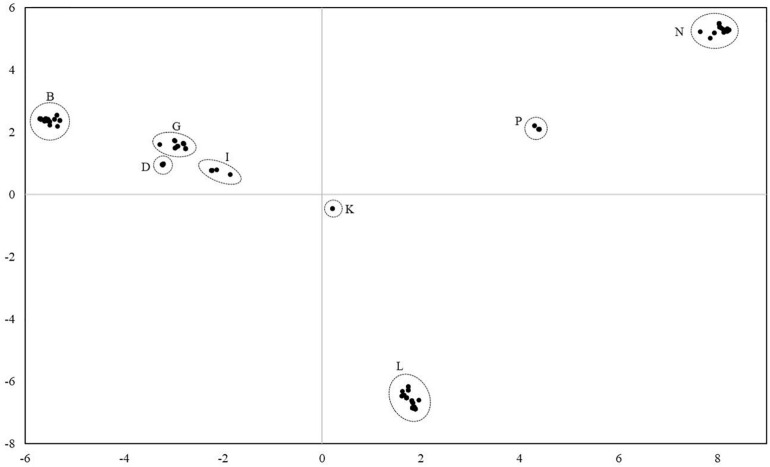
Principal coordinate analysis plot of 271 Holstein mares from 75 lineages considering the mitochondrial genome reveals eight major haplogroups. Haplogroups were named according to Achilli et al. (2012). All mares from one lineage were assigned to the same haplogroup.

For the analysis of the diversity between the lineages, all mares and all lineages were considered. Altogether, 78 distinct haplotypes were identified. They differed from the reference sequence by 34–134 sites. Most of the haplotypes were only represented in a single maternal lineage. In three cases, two lineages shared the same haplotype and in three cases, three different lineages shared the same haplotype. Thus, individuals from different lineages had identical mtDNA sequences. The haplotypes were compared with previously published haplotypes using the BLAST search in the NCBI database. However, no complete homology could be found. On average, they differed from deposited haplotypes in 6.17 positions ranging from two to twelve. Figure 3 illustrates the genetic distances between all individuals. The results revealed that individuals can be assigned to groups with all mares from one lineage belonging to the same group. According to Achilli et al. (2012) the groups could be assigned to the eight haplogroups B, D, G, I, K, L, P, and N.

Standard estimates of the genetic diversity such as the haplotype diversity, the number of polymorphic sites and the nucleotide diversity for each haplogroup and for all mares are shown in Table 1. The number of individuals per haplogroup ranged from 3 to 78 individuals. The number of maternal lineages per haplogroup ranged from 1 (Haplogroup K) to 24 (Haplogroup B). Haplogroup B was found in almost one third of all studied maternal lineages. The number of haplotypes per haplogroup ranged from 1 to 21 and haplogroup B showed the highest haplotype diversity (0.924). Haplogroups G, L, and N had similarly high values (0.881, 0.897, and 0.905). A moderate haplotype diversity with values between 0.526 and 0.654 could be found in haplogroups D, I, and P. As haplogroup K was only represented by one haplotype, the haplotype diversity was zero. The number of polymorphic sites within each haplogroup ranged from zero in haplogroup K to 72 in haplogroup N. In general, the nucleotide diversity was very low. Across all individuals, a value of 0.00431 was estimated and the values for the single haplogroups varied between zero and 0.0097.

**TABLE 1:** Distribution of haplogroups [named according to Achilli et al. (2012)] among Holstein mares from the dataset and estimates of the mitochondrial diversity within haplogroups.

Haplogroup	*N*	*N_L_*	*N_H_*	*Hd*	*S*	*π*
B	72	24	21	0.924	49	0.00042
D	13	4	4	0.526	60	0.00060
G	33	9	11	0.881	30	0.00040
I	14	5	4	0.626	18	0.00022
K	3	1	1	0.000	0	0.00000
L	78	16	19	0.897	39	0.00032
P	11	3	3	0.654	2	0.00005
N	47	13	15	0.905	72	0.00097

*N = number of individuals; N_L_ = number of lineages; N_H_ = number of haplotypes; Hd = haplotype diversity; S = number of polymorphic sites; π = nucleotide diversity.*

When comparing the mtDNA sequences of the haplogroups, group-specific variants could be observed. These variants only occurred in the corresponding group and concurrently in all mares of this group. So far, a total of 117 group-specific variants that split into three variants in haplogroup B, nine in haplogroup D, 15 in haplogroup G, 16 in haplogroup I, 28 in haplogroup K, 19 in haplogroup L, 23 in haplogroup P, and 4 in haplogroup N could be observed. These variants could be used as an identifiable motif for the respective haplogroup. Since only one maternal lineage was represented in haplogroup K, some of the group-specific variants in this haplogroup could just be lineage-specific.

Table 2 provides an overview of further estimates to describe the relationships between the identified haplogroups. The average number of pairwise differences within each haplogroup is shown on the diagonal. In line with the previous results, there were no differences between mares in haplogroup K. The highest values were found in haplogroup N: if two individuals were selected randomly from this group, they differed in 16.49 positions on average, i.e., 0.09% of their total mtDNA sequences. The above diagonal elements provide information on the average number of pairwise differences between the haplogroups ranging from 45.99 to 117.28. Pairwise *F*_ST_ values are presented below the diagonal elements. They ranged from 0.05 to 0.19. The results shown in Figure 3 and Table 2 both indicate that haplogroup B and L are the most distinct haplogroups with a high genetic differentiation. Haplogroup N is also located distantly from the others with an average number of pairwise differences between haplogroups always being over 100, except for haplogroup P with a value of 80.34. In contrast, haplogroup B and D have a low genetic differentiation according to the pairwise *F*_ST_ values with the highest sequence concordance. For all other pairs of groups, the *F*_ST_ values ranged between 0.07 and 0.13, thus, indicating a moderate genetic differentiation.

**TABLE 2:** Relationships between corresponding haplogroups [named according to Achilli et al. (2012)] represented by the *F*_ST_ values and the average number of pairwise differences between and within haplogroups.

	B	D	G	I	K	L	N	P
B	7.01	45.99	63.96	64.36	90.79	78.27	113.36	106.59
D	0.05	10.01	63.12	65.30	87.76	75.40	112.31	105.34
G	0.07	0.07	6.65	64.95	90.01	83.65	114.30	104.86
I	0.07	0.08	0.08	3.59	90.80	80.11	114.54	106.24
K	0.11	0.10	0.10	0.11	0	82.77	117.28	106.65
L	0.19	0.09	0.09	0.09	0.10	5.41	103.67	94.34
N	0.12	0.12	0.13	0.13	0.13	0.11	16.49	80.34
P	0.13	0.12	0.12	0.13	0.13	0.11	0.09	0.76

*Above diagonal: average number of pairwise differences between haplogroups; diagonal elements: average number of pairwise differences within haplogroups; below diagonal: pairwise F_ST_ values.*

The haplogroups shown in Figure 3 were also found using this approach. Furthermore, sub-structures in each haplogroup could be detected. Four separate branches were identified in group B, one of which had split off relatively early. In one of the other branches, there was one major haplotype from which ten other haplotypes have emerged differing from the main major haplotype in one to four nucleotide positions. The haplotypes present in haplogroup D as well as in haplogroup G split into three branches. Haplogroup N appears to have developed from haplogroup P and showed several branches. Haplogroup I showed two main branches. Three main branches were identified in haplogroup L, whereas especially one branch is strongly represented in this study.

## Discussion

### Mitochondrial DNA Sequence Variation

This study is the first analysis of Holstein maternal lineages based on mtDNA sequences and reveals a pronounced mitochondrial variability within the population. Even though not all female lineages with importance for the current population were included in the dataset, the sample covers 75 lineages to which 3,233 mares, i.e., 56.4% of all broodmares from the current breeding population can be assigned. Since the mtDNA sequence is inherited maternally without recombination, it is presumably the same in all mares from a lineage and thus, it would theoretically be sufficient to sequence only one mare per lineage. The results of this study confirm this assumption as very little to no variation was found between mares within a lineage, also suggesting very accurate pedigrees of the examined lineages. This is beneficial for evaluations, where no DNA is available for the individual of interest. Samples from maternal related individuals could then be used instead, as there is a high probability of complete homology.

Mitochondrial DNA sequences from Holstein mares were compared to the reference sequence derived from a Swedish horse (Xu and Árnason, 1994). They differ in 467 positions when considering the whole mitochondrial genome (excluding the repetitive part of the non-coding region). In line with previous studies, this indicates a high level of mitochondrial variability in horses (Lippold et al., 2011; Achilli et al., 2012). 354 substitutions (i.e., 75.8%) have not yet been found in other breeds, 22 of those substitutions were found in all Holstein mares and thus, might be breed-specific variants.

The substitution rate is known to be highly heterogenous across the mitochondrial genome (Ning et al., 2014), which was also found in our study. Considering the size of the protein-coding genes, the *COX* genes comprise the fewest variation with no non-synonymous substitutions in the *COX1* and only one non-synonymous substitution in the *COX2* gene. The highest density of total variants could be found in the *ATP6* gene followed by *ATP8* and *ND6.* Considering only the non-synonymous substitutions, *ATP8* seems to be the most variable gene. For Thoroughbred horses, Yoon et al. (2018) detected similar rankings, with the highest variation in *ND6* and the lowest variation in the *COX* genes. Contrary to this, Harrison and Turrion-Gomez (2006) found no allelic variation in the above mentioned highly variable genes in Thoroughbreds. Additionally, in a sample of East Asian horse breeds, contradicting results regarding the non-synonymous substitutions were found, with low variation in the *ND6* and both *ATP* genes (Ning et al., 2014).

The high density of non-synonymous substitutions in the two *ATP* genes and the *ND6* gene implicate that they may be under positive selection (Kreitman and Akashi, 1995). As the Holstein horse is especially bred for its show jumping ability, these genes could potentially have an influence on their jumping performance. Considering the first half of 2020, the top ten jumping breeds ranked by the WBFSH are: Koninklijk Warmbloed Paardenstamboek Nederland (KWPN), Belgian Warmblood, Selle Français, Zangersheide, Oldenburg Jumping Horse, Holstein, Hanoverian, German Sport Horse, Oldenburg and Westphalian. The Holstein horse has been ranked among the top five jumping breeds for seven times between 2010 and 2020 (World Breeding Federation for Sport Horses, 2020). So far, corresponding analyses of the mtDNA are only available for the Holstein breed, so that a comparison of jumping breeds is not possible. This could explain why many of the variants reported here have not yet been found in other studies.

### Mitochondrial Diversity Within Lineages

In line with previous studies, in which haplotypes are consistent for 80 (Bowling et al., 2000) or more than 200 years (Kavar et al., 1999), respectively, most of the examined mares within a lineage do not differ with regard to their mtDNA sequence. Nonetheless, for 13 of the 75 analyzed lineages, the mares’ mtDNA sequences differed in one to three positions. Some authors interpret the occurrence of more than one haplotype per maternal lineage as incorrect pedigree records (Kavar et al., 2002; Giontella et al., 2020). However, a small number of differences in the nucleotide sequence between mares within a lineage may be more likely caused by *de novo* mutations or alternate fixation of heteroplasmy. Heteroplasmy is the existence of more than one mitochondrial genotype within an individual, which is possible due to the existence of multiple copies of mtDNA within each cell along with its high mutation rate (Bowling et al., 2000). As already reported for other mammalian species, heteroplasmy can segregate into different directions within a few generations (Ashley et al., 1989). Due to the sequencing method used in this study comprising a one-fold coverage of the mitochondrial genome, potential heteroplasmy could not be detected but could be the reason for the observed nucleotide differences.

### Mitochondrial Diversity Between Lineages

Mitochondrial haplotypes from all Holstein mares were compared to assess the diversity between the lineages. Seventy-eight distinct haplotypes could be determined indicating the broad genetic diversity of Holstein maternal lineages. The haplotypes differ from the reference sequence by 34–134 sites and none of them has been described before in other breeds. One reason for this is that investigations of the total mitochondrial genome in horses are rare. Most of the previous studies focused on parts of the non-coding region of the mtDNA, because of its high variability. Analyzing this region is supposed to be sufficient to illustrate the phylogeny of a breed (Kavar et al., 2002). Furthermore, there are no studies about other German warmblood breeds. The predominantly examined breeds were Arabian (Bowling et al., 2000; Jansen et al., 2002) and Lipizzan (Kavar et al., 1999, 2002), as well as small regional breeds. So far, no breed with a close relationship to the Holstein breed has been in the focus of research based on mtDNA sequences.

There is a total of six haplotypes in this study that each occur in two or three different lineages, respectively. Such findings have already been reported in other breeds and is likely a result of closely related or identical ancestral mares (Kavar et al., 1999; Hill et al., 2002). In the Holstein horse, this hypothesis can be checked through inspection of (i) sub-lineages of divided lineages that can be traced back to a common ancestral mare and (ii) the lineage numbers of lineages that share the same haplotype. The latter is due to the fact that at the time of the first recording of pedigree data, lineage numbers were assigned up to 5,000 depending on the region of origin, which affects the majority of the Holstein lineages. Lineages from one region are presumably more closely related than lineages from different regions. Two of the six haplotypes occur in lineages that were on purpose divided into sub-branches when the number of horses belonging to one lineage became too large. Our data set includes one divided lineage with two sub-lineages (318D1 and 318D2 from haplogroup B) and one with three sub-lineages (18A1, 18A2, and 18B1 from haplogroup G). As expected, all related sub-lineages share the same haplotype. The remaining four haplotypes were found in different lineages. Of this, two haplotypes were found in two and three lineages, respectively, with close lineage numbers (95 and 185 from haplgroup I; 42A, 162 and 173 from haplogroup L) that can be assigned to the same region of origin, whereas the other two were found in two and three lineages, respectively, with different regions of origin according to their lineage number (2,581 and 3,401 from haplogroup B; 474A, 1,463 and 8,768 from haplogroup B). As the occurrence of the same haplotype in different lineages indicates common ancestry, these results suggest that there might have been intensive exchange of mares prior to the 19th century, when pedigree recording started in Holstein horse breeding. Thus, lineages from different regions may be more closely related than currently assumed.

### Clustering of Lineages

As shown in Figure 3 mares can be divided into eight clusters corresponding to the haplogroups already identified by Achilli et al. (2012) and based on the genetic distances between their mtDNA sequences. No unknown and, thus, breed-specific haplogroup was identified. Considering the lineage numbers, no geographical pattern could be observed within the haplogroups supporting our assumption of an early maternal gene flow across the former breeding districts. Within a cluster, low genetic distances and low values for the average number of pairwise differences suggest a low variability and, thus, indicates a close maternal relationship between individuals.

The haplotype diversity was highly variable ranging from zero to 0.924. Haplogroup K (Hd = 0.00) is not included in this evaluation due to its low sample size of only three individuals from one lineage. The highest values can be found in haplogroups B, G, L, and N. These haplogroups comprise the highest number of individuals and lineages in this study. Much lower values are present in the remaining haplogroups D, I, and P. In these haplogroups, the haplotype diversity is far below 0.8 indicating a strong founder effect (Cardinali et al., 2016) that possibly could be connected to the history of the Holstein horse breed. After World War II, the number of mares and thus, the number of lineages decreased substantially to one tenth. In 1960, the total population consisted only of around 1,300 mares resulting in a large loss of genetic variation. However, since this data set does not include all maternal lineages of the current population and since the number of mares per lineage or haplogroup is limited, the low values for the haplotype diversity could be due to this limitation. A larger study considering the whole population could provide more information.

**FIGURE 4:**
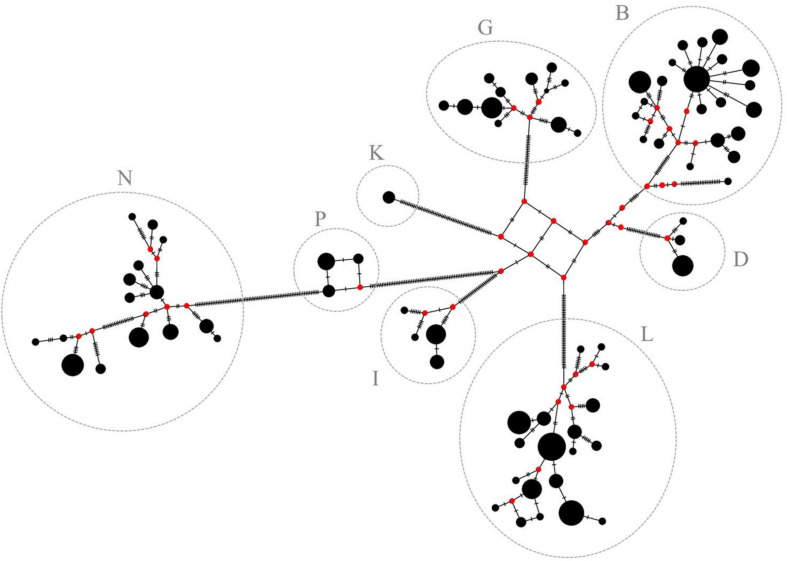
Median-joining network of the 78 haplotypes. Haplotypes are represented by black circles. Size of the circles is proportional to the haplotype frequency. Red circles (median vectors) illustrate missing haplotypes. Haplogroups are assigned according to Achilli et al. (2012) and are in accordance with previous results of this study (see Figure 3 for comparison).

The differences between the haplogroups are much larger than within haplogroups as can be seen from higher values for the average number of pairwise differences between the groups. The haplogroups B, G, I, and D appear to be more closely related to each other than to the remaining haplogroups indicated by the lowest values for the average number of pairwise differences between them in the entire comparison. Haplogroup N seems to be the most distinct one followed by haplogroup P with the highest values for the average number of pairwise differences. The *F*_ST_ values between the haplogroups support the clear clustering of lineages shown in Figures 3, 4.

### Potential Evolution of Lineages

Based on the large similarities of mtDNA sequences within a cluster and the pronounced differences between them, it can be assumed that the mares from one haplogroup can be traced back to a common native ancestral mare that has existed long before pedigrees have been recorded and from which today’s lineages developed. It is not known when the ancestral mare of the respective haplogroups could have existed nor when the individual lineages have differentiated. However, it is possible to illustrate the potential development of haplotypes within the haplogroups using a median-joining network (Figure 4). Median-joining networks are applied to reconstruct intraspecific phylogenies (Bandelt et al., 1999) and have been widely used in mtDNA studies for intra- and interbreed comparisons in horses (Cieslak et al., 2010; Campana et al., 2012; Almarzook et al., 2017). A common ancestral haplotype can be identified within each haplogroup as origin from which all the other haplotypes probably have developed. Hypothesized ancestral haplotypes do not appear to be closely related as can be seen from the high number of strokes between them. Substructures can be found in each haplogroup possibly caused by fixation of *de novo* mutations or heteroplasmy in the population. In haplogroup B, there is a very early branch of a single haplotype that is only represented by one individual and its nucleotide sequence is very different to the others. It could possibly be assigned to a separate haplogroup which, however, cannot be further examined in this study. Additionally, star-like structures can be seen in some haplogroups. Frequent haplotypes (recognizable by the size of the circle) without unique variants are in the center, surrounded by many other haplotypes, which differ by only a few positions of their nucleotide sequence. This can be clearly seen in haplogroup B, but, to some extent, also in haplogroup N. Furthermore, haplogroup N probably has evolved from haplogroup P, possibly concomitant with a common female ancestor. Mares with haplotypes only differing in a few mutations as indicated by the number of strokes, possibly can be consolidated to a common maternal lineage.

## Conclusion

This study examined maternal lineages, to which more than half of all active broodmares can be assigned and provides a comprehensive overview of the Holstein population. We found a high genetic variability of the mtDNA. Some lineages have identical or nearly identical mtDNA sequences and therefore, are most likely closely related on the maternal side. The results indicate that the 75 lineages of the current breeding population included in the dataset have developed from at least eight ancestral founder mares. An investigation of the entire population would possibly reveal further founder mares. Based on our findings, the lineages that were studied could be assigned to consolidated groups. In principle, further studies of the mtDNA sequences of Holstein horses would enable a complete re-definition of the maternal lineages resulting in a much smaller number of female lineages. Pedigree errors were not identified, which indicates a very accurate documentation of the pedigrees. The occurrence of strain- and group-specific variants that have not yet been discovered in other breeds, provides a solid basis and additional information for investigations on the effect of mitochondrial variation on performance traits.

## Data Availability Statement

The datasets presented in this study can be found in online repositories. The names of the repository/repositories and accession number(s) can be found in the article/Supplementary Material.

## Ethics Statement

Ethical review and approval was not required for the animal study because this study is no animal experiment according to the German Animal Welfare Act because hair samples were selected from horse owners during routine care, e.g., combing the mane or tail. This was confirmed by the animal welfare officers at the University of Kiel. Written informed consent was obtained from the owners for the participation of their animals in this study.

## Author Contributions

NK and DB performed the conceptualization of the study. GT did the acquisition of the financial support together with IR. TN provided the data. DB conceived the molecular experiments. LE performed the lab works and analyzed the data in collaboration with NK and DB. LE and NK prepared the final manuscript supported by DB and GT. All authors read and approved the manuscript before submission.

## Conflict of Interest

The authors declare that the research was conducted in the absence of any commercial or financial relationships that could be construed as a potential conflict of interest.

## Footnotes

1 https://www.ncbi.nlm.nih.gov/genbank
